# Reproductive Efficiency of Nelore Cows in Fixed-Time Artificial Insemination Programs with Early Resynchronization

**DOI:** 10.3390/vetsci12010027

**Published:** 2025-01-08

**Authors:** Larissa de Paiva Nunes Gonçalves, Alisson Jordão Prado, Aline Pacheco, Yana Eliza Feitosa de Almeida, Pietro Sampaio Baruselli, Welligton Conceição da Silva, Antônio Humberto Hamad Minervino, Jucelane Salvino de Lima, Kedson Alessandri Lobo Neves

**Affiliations:** 1 Postgraduate Program in Biosciences, Federal University of Western Pará (UFOPA), Santarem 68040-255, PA, Brazil; 2 Postgraduate Program in Animal Science, Federal University of Western Pará (UFOPA), Santarem 68040-255, PA, Brazil; alissonjprado@gmail.com (A.J.P.); jucelane.lima@ufopa.edu.br (J.S.d.L.); 3 Institute of Biodiversity and Forests—IBEF, Federal University of Western Pará (UFOPA), Santarem 68040-255, PA, Brazil; alinepacheco@outlook.com (A.P.); yanaelizafeitosadealmeidaalmei@gmail.com (Y.E.F.d.A.); kedson_neves@hotmail.com (K.A.L.N.); 4 Department of Animal Reproduction, School of Veterinary Medicine and Animal Science, University of São Paulo, São Paulo 05508-000, SP, Brazil; barusell@usp.br; 5 Postgraduate Program in Animal Science (PPGCAN), Institute of Veterinary Medicine, Federal University of Para (UFPA), Castanhal 68746-360, PA, Brazil; 6 Laboratory of Animal Health (LARSANA), Federal University of Western Pará (UFOPA), Rua Vera Paz, s/n, Salé, Santarem 68040-255, PA, Brazil; ah.minervino@gmail.com

**Keywords:** biotechnology, beef cattle, follicular dynamics, hormonal protocol

## Abstract

We aimed to evaluate the reproductive efficiency of Nelore cows in fixed-time artificial insemination (FTAI) programs with early resynchronization. A total of 468 Nelore cows were divided into two experimental groups: R30—conventional resynchronization at 30 days (*n* = 234); R22—early resynchronization at 22 days (*n* = 234). Both groups followed a synchronization protocol using intravaginal progesterone devices combined with the administration of steroids and gonadotropins. The R30 protocol involved resynchronization 30 days after FTAI, while the R22 protocol implemented early resynchronization 22 days after FTAI without prior pregnancy diagnosis. In both groups, one FTAI was followed by two resynchronizations. The results showed similar pregnancy rates between the groups, with the pregnancy rate in the 1st FTAI being 50.85% in the R30 group and 48.72% in the R22 group (*p* = 0.742). During the 1st resynchronization, the rates were 45.22% in the R30 group and 46.67% in the R22 group (*p* = 0.742). The cumulative pregnancy rate after the 1st FTAI, 1st, and 2nd resynchronizations was 80.77% in the R30 group and 82.91% in the R22 group (*p* = 0.643), with no significant difference observed. Follicular dynamics were also monitored, with ovulation occurring in 78.95% of cases in the 1st FTAI, 91.67% in the 1st resynchronization, and 88.98% in the 2nd resynchronization. The mean diameter of the ovulatory follicle was 13.64 ± 3.48 mm, and the largest follicle diameter was significantly greater (14.47 ± 2.76 mm) in cows that ovulated compared to those that did not (11.54 ± 4.24 mm) (*p* < 0.05), reinforcing the association between follicle size and the likelihood of ovulation. These findings indicate that both early and conventional resynchronization protocols perform similarly, with early resynchronization offering advantages in reducing the calving interval. Furthermore, the relationship between follicular diameter and ovulation confirms the importance of follicle size as an indicator of reproductive success.

## Simple Summary

We aimed to evaluate the reproductive efficiency of Nelore cows in fixed-time artificial insemination (FTAI) programs with early resynchronization. During the 1st resynchronization, the rates were 45.22% in the R30 group and 46.67% in the R22 group (*p* = 0.742). The cumulative pregnancy rate after the 1st FTAI, the 1st and 2nd resynchronizations were 80.77% in the R30 group and 82.91% in the R22 group (*p* = 0.643), with no significant difference observed. Follicular dynamics were also monitored, with ovulation occurring in 78.95% of cases in the 1st FTAI, 91.67% in the 1st resynchronization, and 88.98% in the 2nd resynchronization. The findings indicate that early and conventional resynchronization protocols perform similarly, with early resynchronization offering advantages in reducing the calving interval. Furthermore, the relationship between follicular diameter and ovulation confirms the importance of follicle size as an indicator of reproductive success.

## 1. Introduction

Fixed-time artificial insemination (FTAI) is one of the most widely used biotechnologies because it eliminates the need for estrus observation, reduces the calving interval (CI), and improves the genetics of the herd [1,2,3,4].

Resynchronization is widely used in beef cattle farms in order to provide second chances for cows that were diagnosed as nonpregnant after insemination, further increasing the herd’s productivity and efficiency [5]. The use of FTAI combined with resynchronization as a strategic management method makes it possible to reduce the time between inseminations, a smaller number of clean-up bulls, and a higher pregnancy rate at the end of the breeding season [6].

In conventional resynchronization, the diagnosis of pregnancy is made by ultrasonography 30 days post-insemination; at this time, females diagnosed as nonpregnant undergo a new protocol for resynchronization, thus making it possible to obtain an interval of 40 days between inseminations [7]. However, in early resynchronization, on the 22nd day after insemination (regardless of the pregnancy diagnosis), a new FTAI protocol is initiated in all females. On the 30th day, all females are submitted to a pregnancy diagnosis, and only the nonpregnant females follow the FTAI protocol, being inseminated on the 32nd day, i.e., with a shorter interval than conventional resynchronization [8].

Therefore, this study is important as it presents a protocol proposal that can be used by producers in different regions, maximizing the pregnancy rate and, subsequently, calf production. The aim was to evaluate the parameters related to the reproductive efficiency of Nelore cows submitted to the FTAI protocol followed by two early resynchronizations compared to conventional resynchronization.

## 2. Materials and Methods

### 2.1. Ethical Aspects

This study was approved by the Ethics Committee on the Use of Animals (CEUA) of the Federal University of Western Pará under protocol number 0120230238.

### 2.2. Location

The experiment was carried out in a rural property located in the municipality of Mojuí dos Campos, state of Pará (latitude of 2°41′05″ S; longitude 54°38′35″ W).

### 2.3. Animals

A total of 468 Nelore cows (*Bos taurus indicus*) were used in the absence of clinical signs of infectious or metabolic disease and were clinically sound after gynecological examination. The cows were classified according to the reproductive category: primiparous, first-calf females; secundiparous, second-calf females; and multiparous, third-calf females or more. Additionally, the body condition score (BCS) of the cows was assessed at the beginning of each synchronization and resynchronization protocol, using a scale of 1 to 5 (1 being very thin and 5 obese), according to Ayres et al. [9].

During the experimental period, the cows were kept on pastures of *Brachiaria brizantha* cv. Marandu at an adequate stocking rate (one animal unit per hectare). The animals received mineral supplementation, water *ad libitum*, and strict sanitary control.

### 2.4. Experimental Design

The cows were randomly allocated to both experimental groups: conventional (R30, *n* = 234) and early (R22, *n* = 234).

#### 2.4.1. Hormonal Synchronization Protocol

In both groups, the ovulation synchronization protocol was initiated on day 0 (D0) with the insertion of an intravaginal device containing 0.5 mg of progesterone (DIB^®^, Zoetis, São Paulo, Brazil), combined with the intramuscular (IM) administration of 2 mg of estradiol benzoate (EB) (Bioestrogen^®^, Biogénesis Bagó, Curitiba, Brazil). On day 8 (D8), the progesterone device was removed, and 0.4 mg of estradiol cypionate (E.C.P.^®^, Zoetis, São Paulo, Brazil), 2.5 mg of dinoprost tromethamine (Lutalyse^®^, Zoetis, São Paulo, Brazil), and 300 IU of equine chorionic gonadotropin (eCG) (Novormon^®^, Zoetis, São Paulo, Brazil) were administered via IM injection. On the same day, the cows were marked with a marker stick between the sacral tuberosity and the insertion of the tail to assess estrus expression. The animals were kept in the same group, with cows that exhibited signs of estrus having the paint on the tail base removed due to homosexual behavior, which was followed by acceptance of mounting from other cows. Artificial insemination was performed on day 10 (D10), between 48 and 54 h after the removal of the progesterone device. In cows that did not show estrus manifestations (i.e., the paint remained on the tail base), 2.5 mL of GnRH (Gonaxal^®^, Biogénesis Bagó, Curitiba, Brazil) was administered.

#### 2.4.2. Conventional Resynchronization Protocol (R30)

Pregnancy diagnosis (PD) was performed via transrectal ultrasonography (Mindray^®^, DP 50 VET, Shenzhen, China) 30 days after the first insemination (D40). Cows diagnosed as non-pregnant were identified and resynchronized with the same hormonal protocol used in the first artificial insemination (AI), with the second insemination performed on D50. After 30 days, cows that remained non-pregnant after the second AI were subjected to a second resynchronization on D80. The protocol was repeated, with insemination performed approximately on D90. This resynchronization cycle allowed for the performance of three inseminations within 80 days.

#### 2.4.3. Early Resynchronization Protocol (R22)

**Figure 1:**
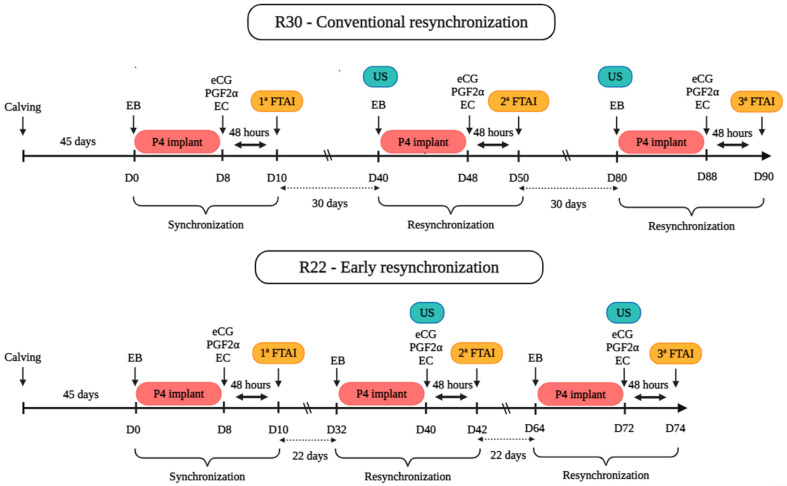
Protocol used in the experimental groups. eCG = equine chorionic gonadotrophin; PGF2α = Prostaglandin F2α; US = ultrasound; EB = Estradiol Benzoate; FTAI = Fixed-time artificial insemination; P4 = progesterone; EC = estradiol cyprionate.

Unlike the conventional approach, where resynchronization occurs only after pregnancy diagnosis, the early resynchronization protocol (R22) was performed without prior pregnancy diagnosis, allowing the cows to be prepared for the second insemination within a shorter period. For early resynchronization, an intravaginal progesterone device (DIB^®^, Zoetis, São Paulo, Brazil) was inserted into the cows on D32, along with the intramuscular (IM) administration of 1 mg of estradiol benzoate (EB) (Bioestrogen^®^, Biogénesis Bagó, Curitiba, Brazil). On day 40 (D40), 30 days after the first insemination, the progesterone implant was removed, and pregnancy diagnosis was performed via transrectal ultrasonography (Mindray^®^, DP 50 VET, Shenzhen, China). Cows diagnosed as non-pregnant continued the hormonal treatment according to the resynchronization protocol, with the progesterone device removed, the marker stick applied, and 0.4 mg of estradiol cypionate (E.C.P.^®^, Zoetis, São Paulo, Brazil), 2.5 mg of dinoprost tromethamine (Lutalyse^®^, Zoetis, São Paulo, Brazil), and 300 IU of equine chorionic gonadotropin (eCG) (Novormon^®^, Zoetis, São Paulo, Brazil) administered via IM injection. The second insemination was performed two days after the removal of the P4 device on D42. In cows that did not express estrus, 2.5 mL of GnRH (Gonaxal^®^, Biogénesis Bagó, Curitiba, Brazil) was administered. Subsequently, on day 64 (D64), 22 days after the second AI, a second resynchronization was performed using the same protocol as before. Pregnancy diagnosis was performed on D72, and the third artificial insemination occurred on D74, thus allowing three inseminations to be performed within 64 days, as shown in Figure 1.

The cows were inseminated with frozen semen from different Nelore bulls from insemination centers and with proven sperm quality. At the time of insemination, semen thawing was performed at 37 °C for 30 s with the aid of a thawing machine (Intragen^®^, São Paulo, Brazil).

### 2.5. Evaluation of Follicular Dynamics to Determine Ovulation Rate, Time of Ovulation, and Follicular Diameter

About 49 cows were randomly chosen for ultrasound examinations in order to verify the ovulation rate, the moment of ovulation, and the follicular diameter.

**Figure 2:**
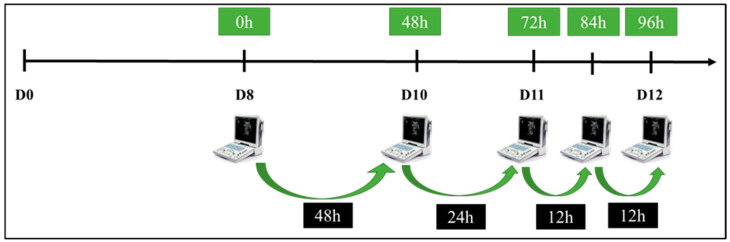
Schematic model of ultrasound examinations to verify the ovulation rate, measurement of follicle diameter, and time of ovulation.

Ultrasonographic assessments were conducted using an ultrasound device (Mindray^®^, DP 50 VET, Shenzhen, China) equipped with a 7.5 MHz linear rectal transducer. The evaluations began on day 8 of each protocol, coinciding with the removal of the intravaginal P4 devices. Subsequent examinations were performed 48 h (D10), 72 h (D11), 84 h (D11), and 96 h (D12) after implant removal (Figure 2).

Ovulation was considered to have occurred when the dominant follicle, previously identified during ultrasonographic examination, was no longer visible on the ovary. Consequently, the timing of ovulation was defined as the moment during examination when the ovulatory follicle disappeared.

Cows that retained an ovulatory follicle until 96 h after implant removal were classified as non-ovulated. Follicles were defined as non-ovulatory if they remained visible in the same location throughout the evaluation period without exhibiting any morphological changes indicative of ovulation.

The diameter of the largest follicle present at the time of intravaginal device removal was measured. Additional measurements of follicular diameter were taken 48 h (D10), 72 h (D11), 84 h (D11), and 96 h (D12) after implant removal.

### 2.6. Statistical Analysis

Statistical analysis was performed using the Statistical Analysis System (SAS^®^) Software (version 6.12). Descriptive statistics and analysis of variance were used to measure the association between pregnancy rate (dependent variable), BCS, experimental groups (R30 and R22), and reproductive category (primiparous, secundiparous, and multiparous). In all analyses, a *p*-value < 0.05 was used for statistical significance. For the variable pregnancy rate, comparisons of frequencies (proportions) were performed using the chi-square test. When a significant effect on ANOVA was detected, Tukey’s test was applied to discriminate the means. The ovulation rate and moment of ovulation were estimated to evaluate the follicular dynamics, and the mean follicle diameter (DFOL) was measured at different measurement times.

## 3. Results

The pregnancy rate (PR) was compared between the two resynchronization protocols, R30 (conventional resynchronization) and R22 (early resynchronization), at different insemination and resynchronization times. In the 1st fixed-time artificial insemination (FTAI), the R30 group had a pregnancy rate of 50.85% (119/234), while the R22 group had 48.72% (114/234), with no statistically significant difference between the groups (*p* = 0.742).

In the 1st resynchronization, the pregnancy rate was 45.22% (52/115) for the R30 group and 46.67% (56/120) for the R22 group, again with no statistically significant difference (*p* = 0.742).

For the 2nd resynchronization, the pregnancy rate was 28.57% (18/63) for the R30 group and 37.50% (24/64) for the R22 group, with no significant difference between the groups (*p* = 0.303).

The cumulative pregnancy rate after the 1st FTAI, 1st resynchronization, and 2nd resynchronization was 80.77% (189/234) for the R30 group and 82.91% (194/234) for the R22 group, with no statistically significant difference (*p* = 0.643).

These results indicate that both resynchronization protocols, R30 and R22, showed similar performances in terms of pregnancy rate under the study conditions, with no significant difference between them (Table 1).

The statistical analysis revealed variations in pregnancy rates among the different body condition scores. In body condition score 4, a significant difference was observed between the two protocols, with a *p*-value of 0.049, where the R30 protocol had a significantly higher pregnancy rate (50%) than the R22 protocol (31.48%). For body condition scores 2.5, 2.75, 3, 3.25, 3.5, and 3.75, no statistically significant difference was found in pregnancy rates between the R30 and R22 protocols, with *p*-values greater than 0.05 (Table 2).

**Figure 3:**
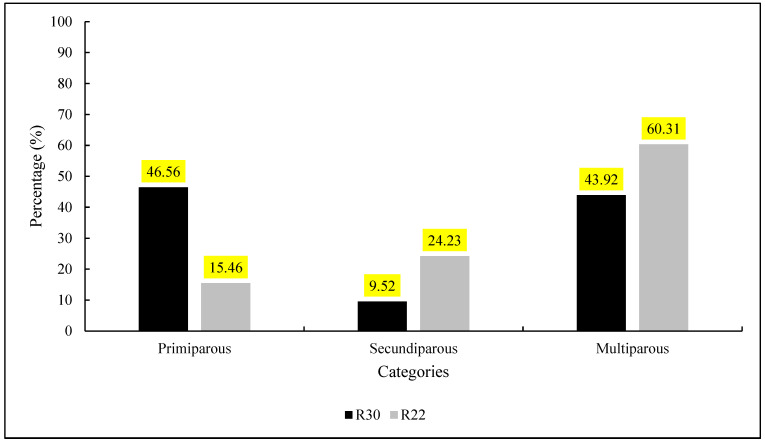
Pregnancy rate according to the reproductive category of females submitted to conventional (R30) and early (R22) resynchronization.

In this study, we compared pregnancy rates between two experimental groups, R30 and R22, in the categories of primiparous, secundiparous, and multiparous. The data showed that, in the R30 group, primiparous cows had a pregnancy rate of 46.56%, while secundiparous and multiparous cows exhibited rates of 9.52% and 43.92%, respectively. On the other hand, the R22 group showed pregnancy rates of 15.46% for primiparous, 24.23% for secundiparous, and 60.31% for multiparous cows (Figure 3).

A statistical analysis was conducted, and the resulting *p*-value was less than 0.001, indicating a statistically significant difference in pregnancy rates between the R30 and R22 groups.

**Figure 4:**
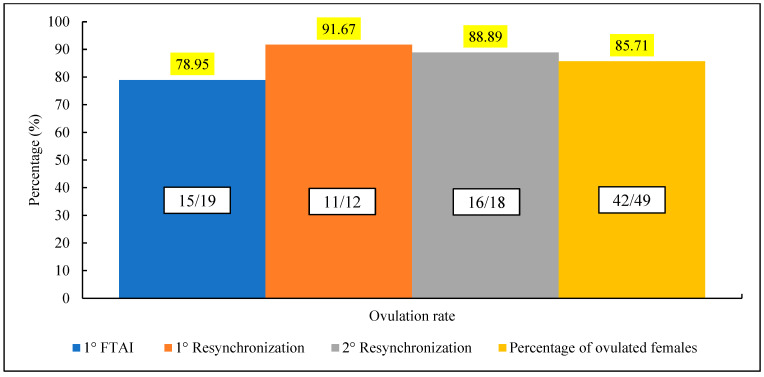
Ovulation rate of females submitted to early resynchronization.

The analysis of follicular dynamics conducted on females (*n* = 49) subjected to early resynchronization revealed the following ovulation rates at different phases of the protocol: 78.95% in the 1st IATF, 91.67% in the 1st resynchronization, and 88.98% in the 2nd resynchronization. Despite the increase in ovulation rate during the first resynchronization, followed by a slight reduction in the second resynchronization, the values remained high at all stages, reflecting the protocol’s effectiveness. Statistical analyses indicated that the ovulation rates across the different phases of the protocol were insignificant (*p* > 0.05). The total number of ovulated females was 85.71% (Figure 4).

Ovulation rates were analyzed as a function of time after the removal of the progesterone implant. Within 48 h (D10) of implant removal, 9.52% of the females ovulated. At 72 h (D11), the highest concentration of ovulations was recorded, with 69.05%. At 84 h (D11), the ovulation rate was 16.67%, while at 96 h (D12), only 4.76% of the females ovulated.

**Figure 5:**
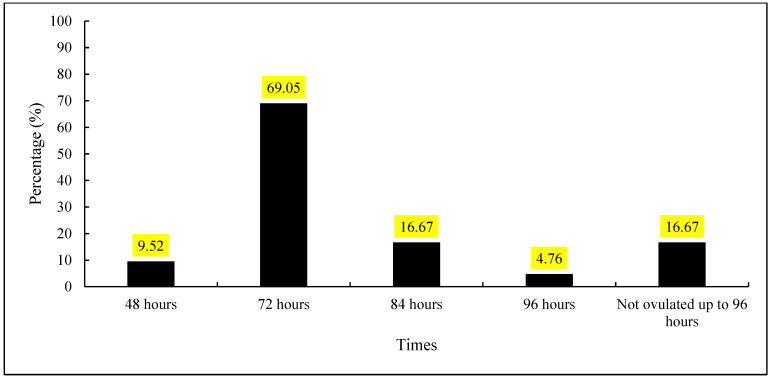
Timing of ovulation of cows after removal of the progesterone implant.

Additionally, 16.67% of the females did not ovulate up to 96 h after implant removal. Statistical analysis revealed a significant difference between the periods (*p* < 0.05), with the ovulation peak occurring at 72 h after implant removal (Figure 5).

The overall mean follicle diameter (DFOL) was 13.64 ± 3.48 mm. The diameter of the largest ovulatory follicle was monitored at different time points after the removal of the progesterone implant. The means and standard deviations observed were as follows: at D10 (48 h), 13.87 ± 3.16 mm; at D11 (72 h), 13.51 ± 3.76 mm; at D11.5 (84 h), 13.59 ± 4.65 mm; and at D12 (96 h), 12.30 ± 4.09 mm. Although the values showed variation over time, the differences between the time points were not statistically significant (*p* > 0.05). The results are presented in Table 3.

The mean diameter of the ovulatory follicle (DFOL) was evaluated in females that ovulated and did not ovulate after the removal of the progesterone (P4) implant, as shown in Table 4. The mean diameter of the ovulatory follicle was significantly larger (*p* < 0.05) in females that ovulated (14.47 ± 2.76 mm) compared to those that did not ovulate (11.54 ± 4.24 mm).

These data reinforce that larger follicles are associated with a higher probability of ovulation, confirming the relevance of follicle size as an indicator of reproductive success in insemination protocols.

## 4. Discussion

The results of this study indicated that the R30 (conventional) and R22 (early resynchronization) protocols exhibited similar performance in terms of pregnancy rates, with no statistically significant differences observed between the groups at different insemination and resynchronization stages.

These findings align with the literature, which highlights the effectiveness of both strategies in optimizing reproductive efficiency in cattle herds. Crepaldi et al. [10] compared cumulative pregnancy rates during a 64-day breeding season across the following treatments: (1) FTAI followed by natural mating, (2) FTAI followed by resynchronization 22 days after the first FTAI and subsequent natural mating, and (3) FTAI followed by two resynchronizations. The authors used 2 mg of estradiol benzoate (EB) in the first FTAI protocol and only 1 mg in the resynchronizations. Pregnancy rates after the first FTAI were similar across the groups. However, cumulative pregnancy rates were higher in groups subjected to one (87.7%) or two resynchronizations (87.8%) compared to the group relying solely on FTAI followed by natural mating (77.1%), demonstrating that using one or two resynchronizations was more efficient than FTAI with natural mating alone.

Campos et al. [11] observed pregnancy rates of 54.4% following the first FTAI and 42.3% after the second FTAI, resulting in a cumulative pregnancy rate of 74.0% within a 33-day interval in lactating Nelore cows resynchronized 23 days after the first FTAI. The protocol used included estradiol benzoate, a progesterone implant, and prostaglandin.

Penteado et al. [12] conducted early resynchronization at 22 days, achieving a pregnancy rate of 48% in the first FTAI and 56% in the resynchronization, with a cumulative pregnancy rate of 77%.

Sá Filho et al. [8] studied the response of beef heifers to resynchronization programs initiated 22 days after AI. The authors reported pregnancy rates of 57.1% for the first FTAI and 61.5% for the second FTAI, leading to an approximate cumulative pregnancy rate of 75% within the first 32 days of the breeding season.

Rodrigues et al. [13] compared pregnancy rates among four different post-FTAI management strategies in a herd of multiparous Nelore cows 45 days postpartum, with an average BCS of 3.7 (scale 1–5). The strategies included continuous exposure to Nelore bulls throughout the breeding season (161 cows), estrus detection with AI (132 cows), conventional resynchronization (157 cows), and early resynchronization (157 cows). After these treatments, the cows were exposed to bulls until the end of the breeding season (75 days). Pregnancy rates for early resynchronization and conventional resynchronization were 45.3% and 46%, respectively. At the end of the breeding season, cumulative pregnancy rates were 98.38% (early resynchronization), 90.62% (conventional resynchronization), 63.30% (natural service control), and 78.95% (estrus detection and AI). These findings demonstrate that resynchronization protocols are more efficient for producing crossbred calves and improving final pregnancy rates.

Sá Filho et al. [8] reported an approximately 75% cumulative pregnancy rate after resynchronizing beef heifers over a 32-day breeding season with two FTAI sessions during this period. These results demonstrate a significant increase in pregnancy rates from resynchronization programs implemented early in the breeding season, highlighting their importance as a tool in enhancing reproductive efficiency in breeding herds. The same authors also observed that synchronizing beef heifers for the first FTAI using either GnRH or estradiol benzoate resulted in similar pregnancy rates (41.5% and 41.9%, respectively). However, resynchronization 22 days post-FTAI using the same hormones favored estradiol benzoate (49.3%) over GnRH (37.2%). They emphasized that administering 1 mg of estradiol benzoate on day 22 post-FTAI did not compromise pre-established pregnancies.

The choice between conventional resynchronization and early resynchronization protocols should consider the cost-benefit ratio and production system objectives. Early resynchronization may be more advantageous in systems prioritizing shorter reproductive intervals, whereas the conventional approach could reduce direct costs associated with hormonal treatments for pregnant females.

Early resynchronization can reduce the calving interval by shortening the time required to identify non-pregnant females and restarting reproductive management. By initiating 22 days post-FTAI, irrespective of pregnancy diagnosis, early resynchronization eliminates the need to wait for traditional pregnancy diagnosis (28–32 days), thereby accelerating the timeline for new insemination. Studies indicate that this protocol minimizes the non-productive period between reproductive failure and subsequent conception. As a result, cows that conceive early can return to the production cycle more efficiently, reducing the calving interval compared to conventional resynchronization protocols, which delay management until after pregnancy diagnosis [12,14].

The body condition score (BCS) is a critical factor influencing reproductive performance in cattle, directly impacting pregnancy rates. The present study revealed significant differences in pregnancy rates among cows with a BCS of 4 between the R30 (conventional) and R22 (early resynchronization) protocols. In this context, the R30 protocol showed a significantly higher pregnancy rate (50%) compared to the R22 protocol (31.48%; *p* = 0.049).

Studies like Pfeifer et al. [15] also observed that Nelore cows with a high BCS (≥4 on a 1-to-5 scale) exhibit lower pregnancy rates per artificial insemination (P/AI). Early resynchronization protocols demand greater hormonal responsiveness over shorter intervals, which can be challenging for cows with elevated BCS due to metabolic and hormonal disturbances that compromise follicular development, ovulation, and pregnancy maintenance [16,17].

Cows with excessive BCS face additional risks, such as estrous cycle irregularities caused by fat accumulation in the reproductive system, increased likelihood of abortion, dystocia, and lower milk production [18]. These factors exacerbate the challenges faced by such cows in subsequent reproductive cycles.

In a second resynchronization, the group mainly comprises cows that failed to conceive previously, potentially representing a subgroup with lower fertility due to metabolic, environmental, or genetic factors, which further reduces reproductive success.

Beyond BCS, differences in reproductive categories should be considered when adopting strategies to enhance reproductive efficiency in cows. In the R30 group, primiparous cows achieved a pregnancy rate of 46.56%, significantly higher than the 15.46% observed in the R22 group. This outcome could be attributed to the greater physiological demands of protocols like R22, which require rapid hormonal responsiveness—a challenge for primiparous cows due to higher nutritional demands for growth and lactation [19].

In secundiparous cows, the R22 group showed better performance, with a pregnancy rate of 24.23% compared to 9.52% in the R30 group. However, pregnancy rates were low in both groups, possibly influenced by factors such as negative energy balance or postpartum metabolic stress. Sartori and Guardieiro [20] highlighted that negative energy balance affects systemic levels of IGF-I, insulin, and glucose, potentially altering LH pulse frequency and thereby impairing follicular growth. Restoring normal LH pulsatility is crucial for the resumption of follicular growth and cyclicity in postpartum cows.

Multiparous cows exhibited the greatest contrast between groups, with pregnancy rates of 43.92% in the R30 group and 60.31% in the R22 group. This result suggests that cows with more reproductive experience may benefit from the R22 protocol, likely due to their higher hormonal responsiveness. According to Grillo et al. [21], there is a notable difference in reproductive performance between primiparous and multiparous cows in many beef cattle systems. Primiparous cows face additional challenges due to the stress of parturition and the combined demands of growth and first lactation, which significantly increase nutritional requirements. These heightened energy demands, especially during periods of pre- or postpartum nutritional restriction, contribute to poorer reproductive responses. In contrast, multiparous cows, having reached full development, do not face the same energy demands for growth, allowing better adaptation to reproductive protocols and, consequently, higher pregnancy rates.

The data indicate that the reproductive category is an important factor to consider when selecting a resynchronization protocol. While R30 appears more suitable for primiparous cows, R22 demonstrated advantages for secundiparous and multiparous cows. These findings emphasize the need to tailor reproductive strategies to the specific reproductive category to maximize pregnancy rates and herd reproductive efficiency.

The results of this study also revealed high ovulation rates at all stages of the early resynchronization protocol, utilizing estradiol cypionate (EC) as an ovulation inducer after the progesterone (P4) implant removal. Ovulation rates were 78.95% in the 1st FTAI, 91.67% in the 1st resynchronization, and 88.98% in the 2nd resynchronization, indicating the efficacy of the inducer.

The use of estradiol esters, such as estradiol cypionate (EC), is widely employed to induce synchronized ovulation in insemination protocols. Previous studies demonstrated that EC, in association with progesterone devices, promotes the emergence of a follicular wave and induces ovulation in 75% of cows [22,23]. The efficacy of EC was also confirmed by Sales et al. [24], who reported ovulation rates of 82.8% in cows treated with cypionate.

Analysis of ovulation timing revealed that 69.05% of ovulations occurred 72 h after the progesterone implant removal, representing the ovulation peak. These results align with the literature, which indicates that the application of estradiol esters (e.g., estradiol benzoate or cypionate) predominantly induces ovulation between 72 and 84 h post-implant removal [22,23].

This study further demonstrated that the mean diameter of ovulatory follicles (DFOL) was 13.64 ± 3.48 mm, with variations observed across different time points after the P4 implant removal. The mean diameters recorded were 13.87 ± 3.16 mm at D10 (48 h), 13.51 ± 3.76 mm at D11 (72 h), 13.59 ± 4.65 mm at D11.5 (84 h), and 12.30 ± 4.09 mm at D12 (96 h). While follicular diameter varied over time, the differences were not statistically significant (*p* > 0.05).

However, a significant difference was observed when follicular diameters were analyzed based on ovulation success. The mean follicular diameter was significantly larger in cows that ovulated (14.47 ± 2.76 mm) compared to those that did not ovulate (11.54 ± 4.24 mm) (*p* < 0.05). These findings reinforce the relationship between follicle size and the likelihood of ovulation, corroborating previous studies that associate larger follicles with higher ovulation rates and reproductive success [25].

According to Cavalieri et al. [26], larger ovulatory follicle diameters result in larger corpora lutea (CL), which in turn produce higher levels of progesterone (P4)—a hormone essential for pregnancy maintenance. Conversely, smaller ovulatory follicles are associated with smaller corpora lutea and reduced progesterone production, which can compromise embryonic development and animal fertility.

## 5. Conclusions

The results of this study indicate that the R30 and R22 protocols demonstrated similar performance in pregnancy rates, with no significant differences observed. Early resynchronization proved effective for multiparous cows, while the conventional protocol was more efficient for primiparous cows. Follicular diameter also played a key role, with larger follicles being associated with higher ovulation rates and reproductive success. These findings highlight the importance of tailoring resynchronization protocols to the individual characteristics of cows, thereby enhancing reproductive efficiency.

## Figures and Tables

**Table 1:** The pregnancy rate of cows submitted to conventional and early resynchronization protocols.

Protocol	1ª FTAI	1° Resynchronization	2° Resynchronization	Cumulative Pregnancy Rate
R30	50.85% ^a^ (119/234)	45.22% ^a^ (52/115)	28.57% ^a^ (18/63)	80.77% ^a^ (189/234)
R22	48.72% ^a^ (114/234)	46.67% ^a^ (56/120)	37.50% ^a^ (24/64)	82.91% ^a^ (194/234)

Values followed by distinct lowercase letters in the same column indicate differences according to the Chi-square test (*p* < 0.05).

**Table 2:** Pregnant females, according to the body condition score (BCS), submitted to conventional (R30) and early (R22) resynchronization.

Body Condition Score (BCS)							
Protocol	2.5	2.75	3	3.25	3.5	3.75	4
R30	49.09% ^a^	40.58% ^a^	48.28% ^a^	43.75% ^a^	56.14% ^a^	68.18% ^a^	50.00% ^a^
R22	47.37% ^a^	37.68% ^a^	42.02% ^a^	41.14% ^a^	54.17% ^a^	59.62% ^a^	31.48% ^b^

^a,b^: Values followed by distinct lowercase letters in the same column indicate differences according to the Chi-square test (*p* < 0.05).

**Table 3:** Mean and standard deviation (SD) of ovulatory follicle diameter (DFOL) after progesterone implant removal.

Time Interval After P4 Implant Removal	Diameter of Ovulatory Follicle (DFOL) Mean ± SD (mm)
48 h (D10)	13.87 ± 3.16 ^a^
72 h (D11)	13.51 ± 3.76 ^a^
84 h (D11)	13.59 ± 4.65 ^a^
96 h (D12)	12.30 ± 4.09 ^a^

Values followed by distinct lowercase letters in the same column indicate differences according to the Tukey test (*p* < 0.05). SD = Standard deviation. P4 = Progesterone.

**Table 4:** Average and standard deviation (SD) of ovulatory follicle diameter (DFOL) of ovulated and non-ovulated females.

Variables	N	DFOL Average ± SD (mm)
Ovulated	42	14.47 ± 2.76 ^a^
Not ovulated	7	11.54 ± 4.24 ^b^

^a,b^: Values followed by distinct lowercase letters in the same column indicate differences by the *t*-test (*p* < 0.05).

## Data Availability

Data are contained within the article.
